# Interpretable deep learning for diagnosis of fungal and acanthamoeba keratitis using in vivo confocal microscopy images

**DOI:** 10.1038/s41598-023-35085-9

**Published:** 2023-06-02

**Authors:** Mahmoud Essalat, Mohammad Abolhosseini, Thanh Huy Le, Seyed Mohamadmehdi Moshtaghion, Mozhgan Rezaei Kanavi

**Affiliations:** 1 Department of Electrical and Computer Engineering, University of California, Los Angeles, 56-125B Engineering IV Building, UCLA, 420 Westwood Plaza, Los Angeles, CA 90095-1594 USA; 2 Ocular Tissue Engineering Research Center, Research Institute for Ophthalmology and Vision Science, Shahid Beheshti University of Medical Sciences, No.23, Paidarfard St., Boostan 9 St., Pasdaran Ave., Tehran, 1666673111 Iran; 3 Department of Confocal Scan, Central Eye Bank of Iran, Tehran, Iran; 4 Department of Computer Science, University of California, San Diego, CA USA

**Keywords:** Biomedical engineering, Corneal diseases, Medical imaging

## Abstract

Infectious keratitis refers to a group of corneal disorders in which corneal tissues suffer inflammation and damage caused by pathogenic infections. Among these disorders, fungal keratitis (FK) and acanthamoeba keratitis (AK) are particularly severe and can cause permanent blindness if not diagnosed early and accurately. In Vivo Confocal Microscopy (IVCM) allows for imaging of different corneal layers and provides an important tool for an early and accurate diagnosis. In this paper, we introduce the IVCM-Keratitis dataset, which comprises of a total of 4001 sample images of AK and FK, as well as non-specific keratitis (NSK) and healthy corneas classes. We use this dataset to develop multiple deep-learning models based on Convolutional Neural Networks (CNNs) to provide automated assistance in enhancing the diagnostic accuracy of confocal microscopy in infectious keratitis. Densenet161 had the best performance among these models, with an accuracy, precision, recall, and F1 score of 93.55%, 92.52%, 94.77%, and 96.93%, respectively. Our study highlights the potential of deep learning models to provide automated diagnostic assistance for infectious keratitis via confocal microscopy images, particularly in the early detection of AK and FK. The proposed model can provide valuable support to both experienced and inexperienced eye-care practitioners in confocal microscopy image analysis, by suggesting the most likely diagnosis. We further demonstrate that these models can highlight the areas of infection in the IVCM images and explain the reasons behind their diagnosis by utilizing saliency maps, a technique used in eXplainable Artificial Intelligence (XAI) to interpret these models.

## Introduction

The cornea is the transparent, outermost layer of the eye, covering the iris, pupil, and anterior chamber, and it plays a crucial role in protecting internal eye structures and in light refraction^1^. This area can be damaged through injury, external particles, or pathogenic microorganisms, causing inflammation and swelling in the cornea, which is referred to as keratitis^2^. Infectious keratitis is the swelling and damage of the cornea caused by infectious organisms such as bacteria, viruses, fungi, or parasites, and may lead to various serious complications or blindness without early diagnosis and treatment^3^. Fungal keratitis (FK) is the most devastating and the leading cause of vision loss, with one million cases of blindness caused by FK worldwide, every year^4,5^. Acanthamoeba keratitis (AK) is a rare but highly severe form of keratitis caused by the parasitic acanthamoeba genus of amoebae, which is often associated with the use of soft contact lenses^6^. Diagnosis of AK often proves challenging and delayed, and the disease is difficult to treat with conventional medications, which can lead to permanent visual impairment or blindness^7^.

In Vivo Confocal Microscopy (IVCM) is an emerging non-invasive tool for capturing high-definition cross-sectional images of eye structures, and is of great assistance for early diagnosis of different types of keratitis. It offers significant advantages in terms of accuracy and sensitivity of diagnosis, particularly in detecting deep stromal infections that are not easily diagnosed through corneal scraping. However, this comes at the cost of an increased workload on medical staff and the need for specialized training to interpret IVCM images^8,9^.

Deep learning (DL) is a class of advanced machine learning techniques, in which convolutional neural networks (CNN) can be used to develop sophisticated artificial intelligence (AI) systems to process high-definition data, such as images, quickly and accurately^10^. In image processing applications, CNNs have multiple convolutional layers capable of capturing high-level features of the image into multiple abstraction levels for processing which improves accuracy and versatility, at the cost of requiring a large amount of annotated data^11^. In recent years, the emergence of DL and CNN has proven tremendously useful in medical data analysis and medical image recognition^12–14^. In ophthalmology, multiple DL-based AI systems have been developed for diagnosis of glaucoma and macular degeneration^15,16^. While DL-based systems have proven useful in various medical diagnosis applications, the development of DL-based systems for keratitis, especially in IVCM imaging technology, has been lackluster. Although some studies have developed DL models for the diagnosis of keratitis using slit-lamp biomicroscopy, research on diagnostic AI models using IVCM images remains limited^17–19^. Researchers have developed models on datasets with a limited number of either data samples (as in^17^ with 137 images) or classes (as in^18^ with two classes of healthy and FK images introduced) or patients (as in^19^ based on a small sample of six patients).

The aim of this study is to present a more advanced deep learning model that provides automated diagnostic assistance for different types of infectious keratitis, offering a comprehensive approach to diagnosis when interpreting IVCM images, both for experienced and inexperienced eye-care practitioners. We consider the classification of four types of IVCM images in our study, consisting of fungal keratitis (FK), acanthamoeba keratitis (AK), non-specific keratitis (NSK), and healthy images. NSK refers to other types of inflammations or injuries not induced by fungal, amoebic or bacterial organisms (further details in "Dataset" section). To achieve this, we introduce the IVCM-Keratitis dataset, consisting of 4001 IVCM images along with their official diagnoses. We trained multiple recent DL models and optimized them to find the best model with the highest performance. To the best of our knowledge, this is the first attempt to develop an automated keratitis deep learning model using IVCM images at this scale and class diversity. Furthermore, this is the first model capable of detecting AK cases using IVCM images.

However, although there have been some efforts to interpret DL models in the emerging field of eXplainable Artificial Intelligence (XAI)^20^, DL models developed using IVCM images are still treated as a black box. We also demonstrate the interpretability of deep learning models for IVCM images and its utility to eye-care practitioners in making decisions faster and with more confidence. We used saliency maps^21^ as a tool to show the areas in the images that a DL model would consider significant for its diagnosis.

## Materials and methods

**Figure 1:**
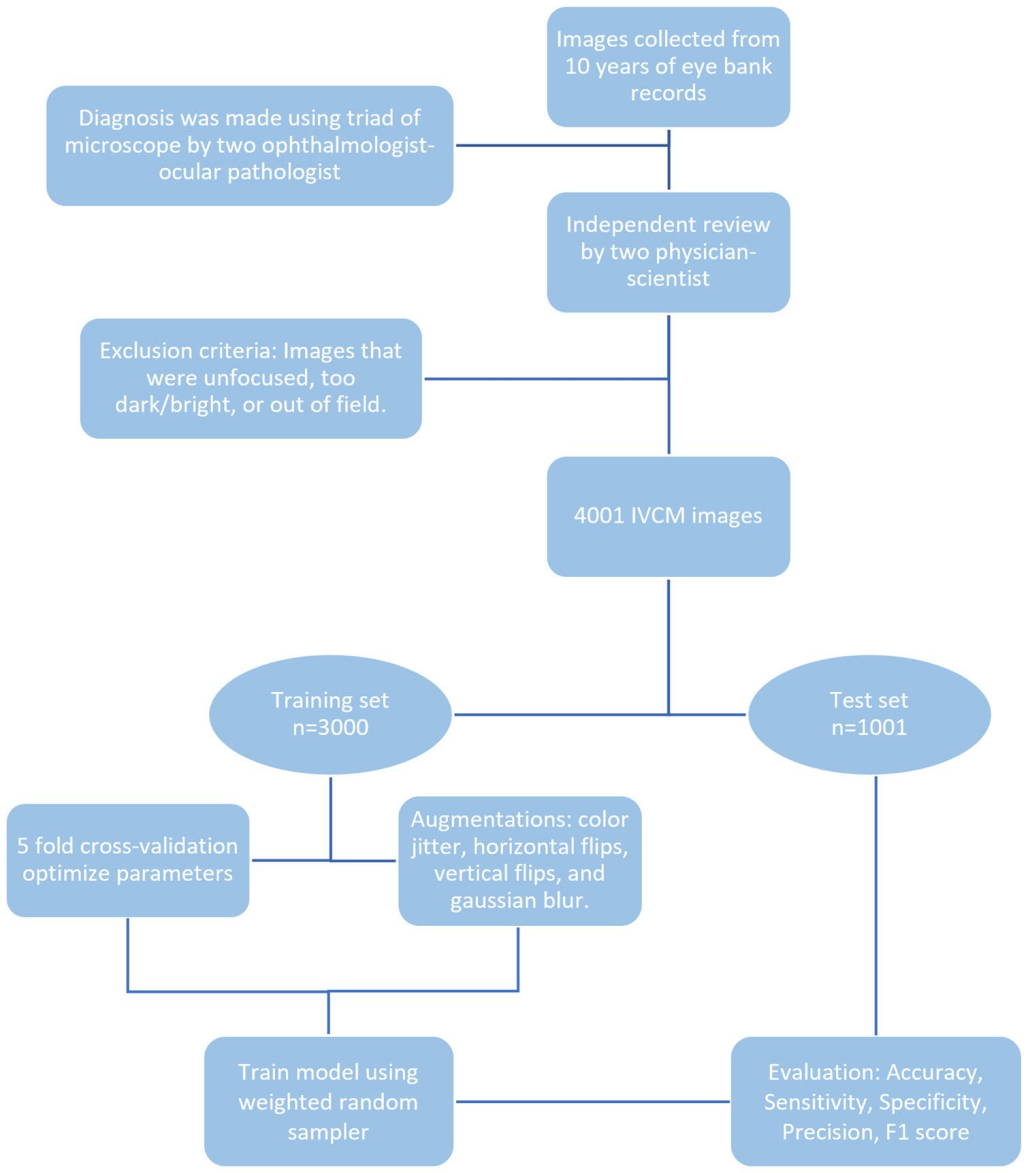
Study pipeline including data collection, training and evaluation processes.

Figure 1 shows the overall pipeline of our study including data collection, training, and evaluation processes. All building blocks will be discussed in the next subsections.

### Dataset

To conduct this study, a full ethical approval was obtained from the ethics committee of the Research Institute for Ophthalmology and Vision Science, Shahid Beheshti University of Medical Sciences, Tehran, Iran (ethical approval code number #IR.SBMU.ORC.REC.1389.20). This retrospective study followed the tenets of the declaration of Helsinki regulations.

The IVCM-Keratitis dataset introduced and used in this paper was gathered from a data pool of IVCM images collected from hundreds of patients who visited the Confoscan unit of the Central Eye Bank of Iran between January 2008 and September 2018. These images were captured using the confocal scan unit ConfoScan 3.0 (Nidek Technology, Padova, Italy).

All the investigated IVCM images had already been confirmed by the microbiologic smear/culture results. Their corresponding clinical features were also consistent with the gold standard of microbiologic smear and cultures (see Supplementary Fig. S1 and Supplementary Fig. S2)^22^.

In order to select the confocal scan images that best represented the diagnosis, two physician-scientists (MA and SMM) independently reviewed all the IVCM images taken during the patients’ visits, and excluded the images that were unfocused, too dark/bright, or out of focus.

**Figure 2:**
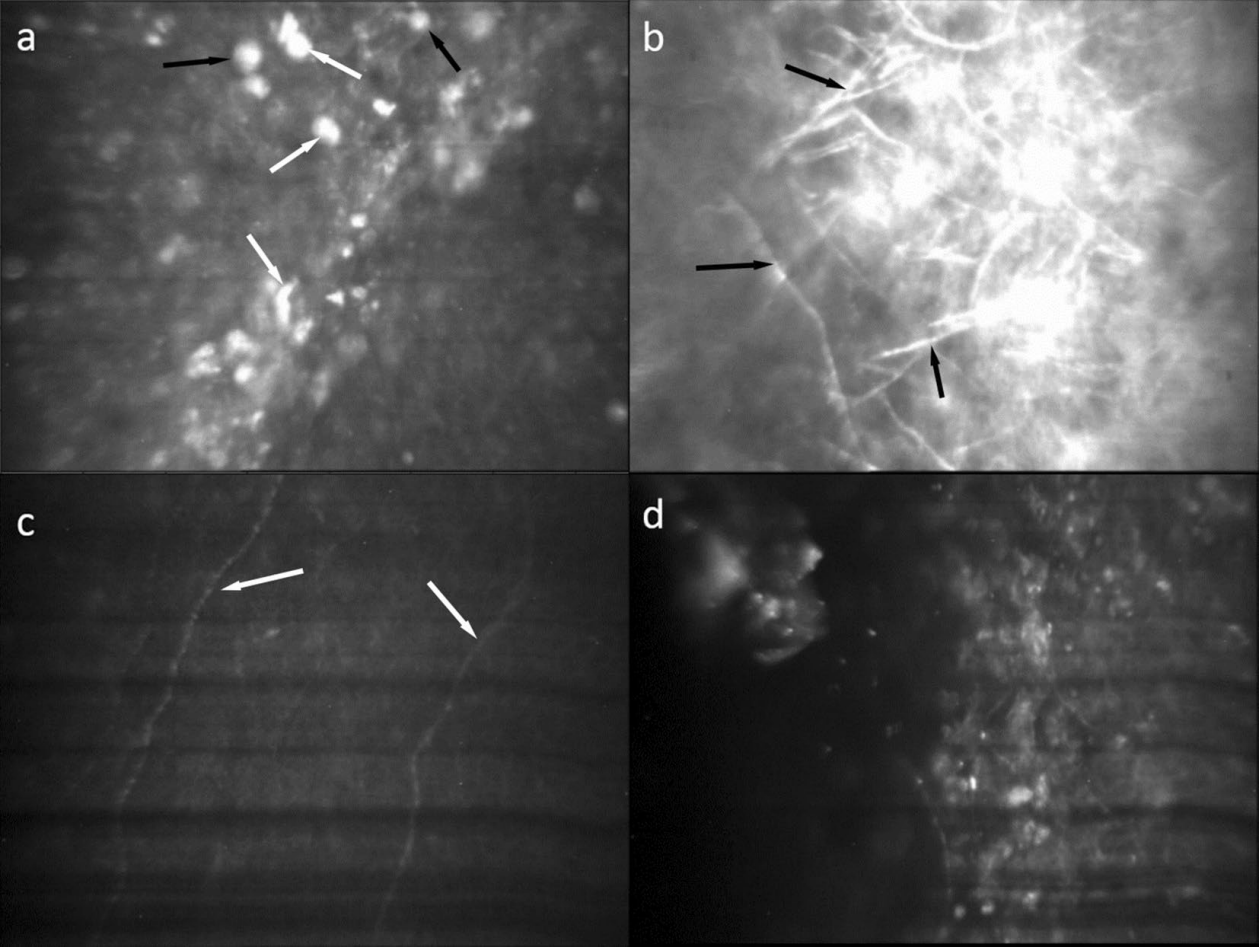
Sample IVCM images of each class showing their different confocal microscopic features. (**a**) Acanthamoeba keratitis, showing acanthamoeba cyst (black arrows) and trophozoite (white arrows) along with inflammatory cells. (**b**) Fungal keratitis, showing fungal filaments (black arrows). (**c**) Healthy cornea, showing normal epithelium along with subepithelial nerves (white arrows). (**d**) Non-specific keratitis, showing hyper reflective structures mainly consisting of inflammatory cells.

The IVCM-Keratitis dataset consists of 4001 IVCM images of size 768 × 576 pixels in JPEG format including 1391 AK, 897 FK, 1004 NSK, and 743 healthy images. The dataset is randomly split into 3000 training and 1001 test images. Sample images of each class are shown in Fig. 2.

The IVCM-Keratitis dataset is made publicly available for research use in the Figshare repository in^23^.

### Data processing

#### Preprocessing

Images were first converted from RGB to grayscale and then normalized to have pixel values between zero and one as shown in Eq. (1).

1 $$y = \frac{1}{256}* \frac{{x}_{r}+{x}_{g}+{x}_{b}}{3}$$

Here, $y$ represents the normalized grayscale pixel values and ${x}_{r}$, ${x}_{g}$, and ${x}_{b}$ represent the pixel values of red, green, and blue channels, respectively.

After that, the mean and variance of the whole dataset were calculated, and each image was standardized relative to the dataset’s mean and standard deviation, as demonstrated in Eqs. (2)–(4).

2 $${\widetilde{y}}^{i}= \frac{{y}^{i}-{\mu }}{\sigma }$$

3 $$\mu =\frac{1}{M*N*|D|} {\Sigma }_{i\in D}{\Sigma }_{m}{\Sigma }_{n} {y}_{mn}^{i}$$

4 $$\sigma =\sqrt{\frac{1}{M*N*|D|} {\Sigma }_{i\in D}{\Sigma }_{m}{\Sigma }_{n}({y}_{mn}^{i}-{\mu }^{i}{)}^{2}}$$

Here, ${y}_{mn}^{i}$ represents the value of the pixel located at the ${m}{th}$ row and ${n}{th}$ column of the ${i}{th}$ normalized grayscale image of size M by N. $\mu$ and $\sigma$ are the mean and standard deviation of our dataset $D$, which has a size of $|D|$.

#### Augmentation

Since deep learning (DL) usually requires a large amount of annotated data, data augmentation methods have been devised to expand the size of the training set. During augmentation, a transformation is applied to the data samples, resulting in new data samples with the same label. Image augmentation methods include mirroring, translation, blurring, zooming, rotation, and others. These methods have been shown to increase a model’s prediction accuracy^24^.

In this paper, we have selected four augmentation methods: color jittering, horizontal flips, vertical flips, and light Gaussian blur. Although data augmentation is typically performed before training, in our study, we have implemented data augmentation dynamically during the training process. For each training epoch, there is a probability of transformation (we set it to 40% in our study) for each image before it is fed into the training process. This approach allows the same image to appear differently during training, effectively augmenting the training set without artificially increasing its size. Moreover, this method enables deep learning models to be trained on large datasets without memory issues.

### Model development

#### Model types

In recent years, DL has grown rapidly with the range of available models greatly expanding. In this study, we trained eight different CNN models that have been extensively used in image classification in recent years, and compared their performance. These models include Densenet161, Densenet121^25^, Resnet152, Resnet101^26^, Resnext101, Resnext50^27^, Cspresnet50^28^, Vgg19, Vgg16, and Vgg13^29^. We did not use the pre-trained weights available for these models, and instead, trained them from scratch with random weight initialization. Specifically, we initialized the linear layers using a uniform distribution within the range of [− 1/|W|, 1/|W|], where |W| represents the output size of the linear layer. For the convolutional layers, we used Xavier initialization^30^.

#### Training

To find the best hyperparameters for each model, such as batch size and learning rate, we performed fivefold cross-validation^31^. In this process, we randomly split the training set into five folds and trained the network on four folds while evaluating its performance on the remaining one-fold. We repeated this process for five rounds to test all the folds, and then calculated the average performance over all the rounds. We trained the model for 10 epochs in each round. Finally, we selected the hyperparameters that led to the best cross-validation performance as the best hyperparameters for the corresponding model.

Using the best hyperparameters for each model, we trained each model for 250 epochs using the training set. We also employed a weighted random sampler during training to deal with any imbalances in the dataset. This sampler samples smaller classes more frequently than larger classes, ensuring that samples from smaller classes appear as many times as samples from larger classes during training.

### Evaluation

To evaluate the performance of our trained models, we used the test set. We report the performance of our models by calculating several performance metrics, including accuracy, precision, recall, specificity, and F1 score. These performance metrics are defined for a two-class classification problem (positive and negative) as follows in Eqs. (5)–(9).

5 $$ACC(\%) = \frac{TP+TN}{P+N}*100$$

6 $$TPR(\%) = \frac{TP}{TP+FN}*100$$

7 $$PPV(\%) = \frac{TP}{TP+FP}*100$$

8 $$TNR(\%) = 2\frac{TN}{N}*100$$

9 $$F1(\%) = 2\frac{PPV*TPR}{PPV+TPR}*100$$

Here, $ACC$, $PPV$, $TPR$, $TNR$, and $F1$ represent accuracy, positive predictive value (precision), true positive rate (recall/sensitivity), true negative rate (specificity), and F1 score, respectively. $P$ represents the number of positive samples, and $N$ represents the number of negative samples. $TP$ (True Positive) is the number of positive samples that are correctly classified, $FP$ (False Positive) is the number of samples that are wrongly predicted as a positive class, and $TN$ (True Negative) and $FN$ (False Negative) are the equivalent of $TP$ and $FP$ for the negative class.

To address the imbalance in the sizes of different classes in the dataset, we calculated the weighted average of these metrics across all classes, using the size of each class as its corresponding weight. Specifically, for each class, samples from that class were considered positive samples, and samples from all other classes were considered negative samples.

## Results

The performance of all 10 trained models is summarized in Table 1. To maintain brevity, we only included the F1 score in this table as it provides a more comprehensive measure of the models’ performance, calculating the harmonic mean of the sensitivity/recall and precision values. Please refer to Supplementary Table S1 for additional details on the performance of the models by other metrics.

**Table 1:** Detailed metrics of the best performing model, Densenet161; accuracy, sensitivity, specificity, precision, and F1 score, along with other models' F1 scores for each class are shown.

Model	Stat	AK	FK	Healthy	NSK	Weighted average
Densenet161	Accuracy	95.70%	96.50%	98.90%	95.90%	96.93%
	Sensitivity	91.37%	96.98%	99.49%	88.79%	94.77%
	Specificity	98.25%	96.38%	98.75%	98.05%	97.80%
	Precision	96.86%	86.94%	95.17%	93.21%	92.52%
	F1	94.04%	91.69%	97.28%	90.95%	93.53%
Densenet121	F1	92.16%	90.23%	84.97%	83.53%	87.29%
Cspresnet50	F1	87.99%	85.71%	78.53%	79.67%	82.42%
Resnet101	F1	92.03%	88.37%	80.0%	76.95%	83.59%
Resnet152	F1	89.71%	85.52%	93.37%	83.58%	88.01%
Resnext101	F1	84.03%	78.46%	52.24%	63.32%	67.76%
Resnext50	F1	86.27%	82.99%	84.21%	76.44%	82.22%
Vgg19	F1	90.42%	84.68%	98.72%	87.98%	90.57%
Vgg16	F1	88.74%	82.0%	96.89%	86.32%	88.56%
Vgg13	F1	91.21%	89.66%	98.48%	88.94%	92.33%

**Figure 3:**
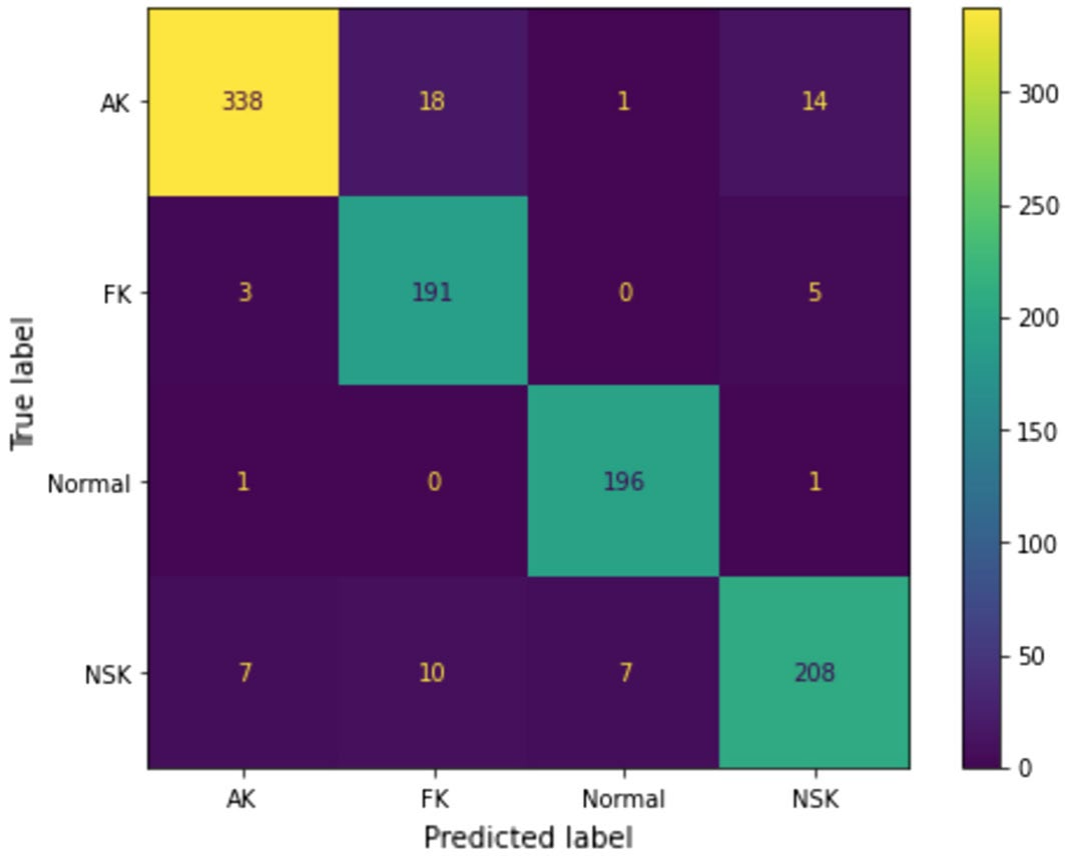
Confusion matrix of Densenet161 applied on the test set, showing the number of mislabeled images for each class. Non-specific keratitis (NSK) had the highest mislabeling rate at 10.3%, followed by Acanthamoeba keratitis (AK) at 8.9%, fungal keratitis (FK) at 4%, and normal at 1%.

Of all the 10 trained models, Densenet161 achieved the best performance with respect to all the metrics, with a weighted average F1 score of 93.53% (Table 1). We summarized the predictions of Densenet161 on the test set in the confusion matrix in Fig. 3. Each cell in the matrix represents the number of samples that have the corresponding predicted and true label. As shown, NSK class has been the most challenging to classify correctly, as it can exhibit similarities with the healthy, AK, and FK classes.

In order to increase the model utility as an aide for practitioners, we implemented a model interpretation method called saliency map, also known as pixel attribution, or gradient-based attribution. A saliency map is generated by calculating the absolute value of the gradient of the output class of a trained model with respect to the input image. This gradient tells us how much the change of any pixel value of the input image will change the classification probabilities on the output classes. In other words, the gradient of each pixel of the input image can tell us how much the model relies on that pixel for prediction, or how important that pixel is to the classification output. By visualizing this gradient, we can see the areas that the model more focused on for its predictions; therefore, giving practitioners more information about how the model made its predictions, which then can be pondered to judge the validity of the predictions. Saliency maps have the advantage of being fast to compute and having the same resolution as the input image^21^.

**Figure 4:**
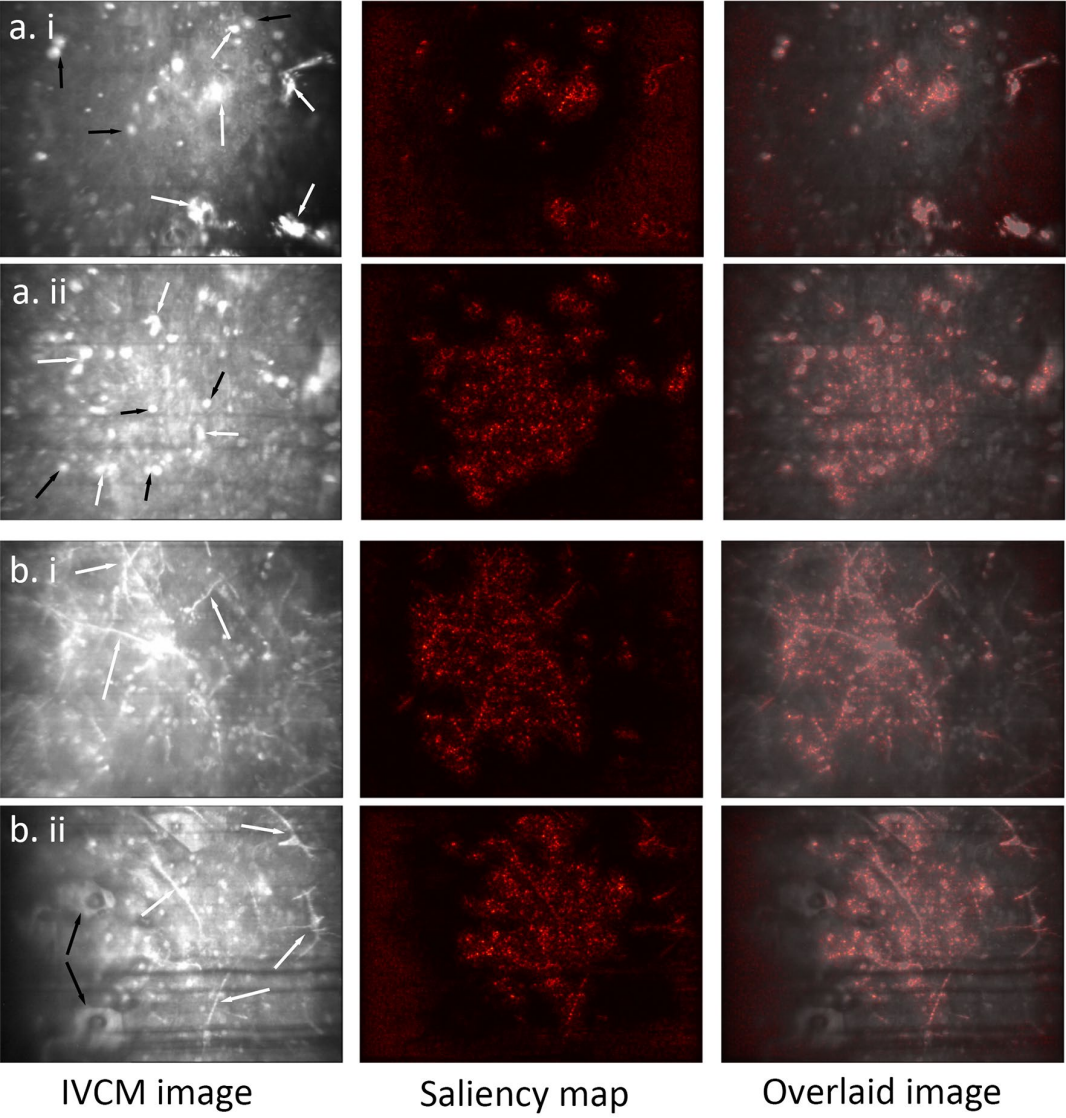
IVCM image samples along with their saliency maps and their overlaid images, displaying different confocal microscopic features. (**a**) Acanthamoeba keratitis, showing acanthamoeba trophozoites (white arrows) and cysts (black arrows), as well as inflammatory cells; (**b**) Fungal keratitis, featuring fungal hyphae (white arrows) and inflammatory cells. Notably, the model correctly ignores superficial cells (black arrows).

**Figure 5:**
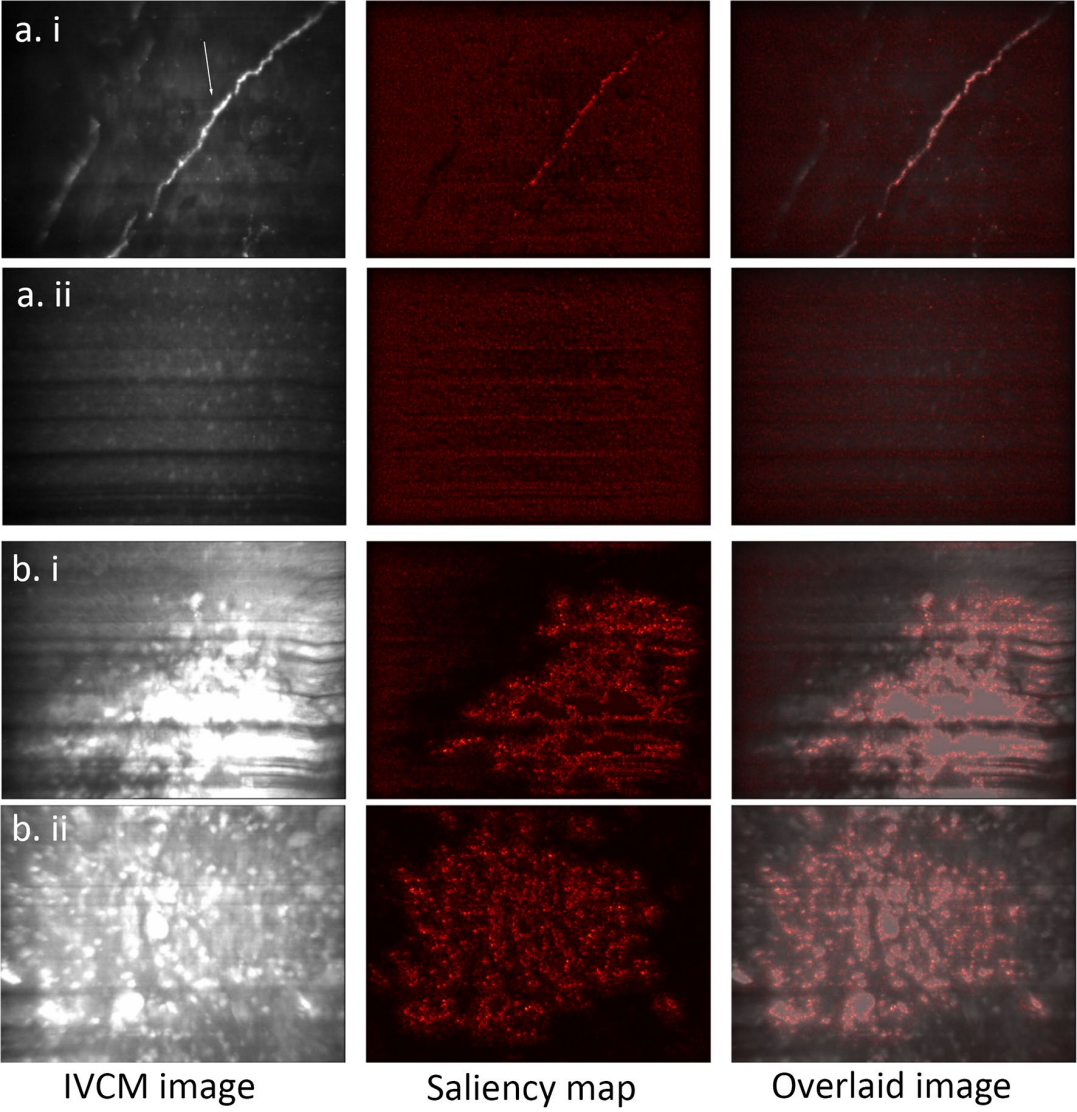
IVCM image samples along with their saliency maps and their overlaid images, displaying different confocal microscopic features. (**a**) Normal cornea, with the model barely considered central corneal nerve (white arrow) as a significant structure, as the saliency map is spread out to the whole image; (**b**) Non-specific keratitis, with the saliency map hardly focusing on any particular structure.

From our analysis of saliency maps to identify the areas that the DL model deems important for diagnosis, we observed that it mainly pays focuses on significant cell structures, such as pathogens and inflammatory cells, while deliberately ignoring the irrelevant structures, such as image artifacts and isolated cells from other layers of ocular surface or tear film (see Fig. 4). However, since there are no peculiar structures in normal, healthy corneal images, the model extends its attention to the entire image. Additionally, as far as we observed, in the test set, the corneal nerve in normal images was never confused with fungal filaments, which can sometimes be misidentified by confocal practitioners, resulting in errors in diagnosis (see Fig. 5).

## Discussion

Amongst the various forms of keratitis, acanthamoeba keratitis (AK) and fungal keratitits (FK) remain the most challenging, as early detection is crucial for effective treatment and better outcomes. Despite advances in laboratory tests, microbiological examinations are time-consuming and have low sensitivity. Although molecular tests are sensitive, they are expensive and require advanced equipment, making image-based diagnosis a more viable option for faster, more accurate, and less demanding diagnoses^32^. However, IVCM image diagnosis of keratitis requires specialized training, which most eye-care practitioners lack^9^. In addition, the time-consuming process of assessing large numbers of IVCM images can lead to delays and inaccuracies in diagnoses^33^. To address this, we introduce IVCM-Keratitis, a large dataset of IVCM images of different types of keratitis, including the less prevalent AK. We also propose a framework for AI-assisted diagnosis of these types of keratitis with high accuracy, using recent Deep Learning (DL) models. Our study shows the interpretability power of these models in indicating the location of the infection in the IVCM images.

During the process of capturing IVCM image, hundreds of images are taken from various layers of the cornea, but most of them are of poor quality or show healthy cells, with only a few displaying infectious structures^34^. This means that physicians would have to manually examine a large number of images containing healthy cells to find the ones that show infections. By training DL models to recognize different types of keratitis and healthy images, these models can quickly sort through a large number of IVCM images. The models can recognize and exclude healthy images, providing eye-care practitioners with images that may show infectious structures, along with the model’s diagnosis and its confidence level. This process can significantly reduce the workload of ophthalmologists and IVCM technicians. Since these models can be trained on NSK images, they can identify difficult images that require accurate diagnosis, which can then be presented to experienced specialists for further evaluation. Having a model that not only provides a diagnosis with its corresponding confidence level but also highlights the areas in the image that led to its diagnosis, can greatly reduce the time needed to assess IVCM images and help eye-care practitioners reach a more accurate conclusion. Although the proposed DL model offers significant benefits in terms of enhancing the accuracy and efficiency of infectious keratitis diagnosis, it is important to emphasize that this model is not intended to replace the experience of skilled eye-care practitioners. Instead, our objective is to provide an additional resource to support their existing expertise and ease the pressure of workload and time constraints they face.

Nevertheless, we do believe that this model has the potential to serve as an adjunct to the diagnostic process by providing a second opinion for less experienced eye-care practitioners and boosting their confidence in their initial diagnosis. This is particularly important because previous research has shown that inexperienced eye-care practitioners generally have lower sensitivity and specificity rates (59% and 92.7% for AK and 42.9% and 87.5% for FK, respectively)^35,36^ compared to our proposed model (91.4% and 98.3% for AK and 97.0% and 96.4% for FK, respectively). Therefore, our model has the potential to improve the accuracy of the diagnosis, while also reducing the risk of errors and misdiagnosis, ultimately leading to better patient management.

We believe that incorporating saliency maps as a model interpretability tool, will greatly benefit practitioners and specialists in improving the promptitude and accuracy of their diagnosis when using IVCM. By highlighting the important regions of medical images, these tools can reduce the workload of practitioners and provide a second opinion in the form of an AI model diagnosis with confidence level.

In general, deep learning requires a significant amount of annotated data to achieve high performance. Although augmentation methods can improve the model performance on smaller datasets, models still benefit greatly from having more manually annotated data. However, obtaining a sufficient amount of annotated data can be challenging for rare diseases, which can cause an imbalance in the dataset. As a result, trained models may be biased towards predicting more prevalent diseases. This is the case for AK, which does not have enough samples in IVCM image databases. In this study, we present an almost balanced, high-definition dataset that includes 1391 images of AK. To the best of our knowledge, this is the first time that AK has been included in a diagnostic system using IVCM images.

While our study has a large dataset, it only includes distinct images selected by specialists, which may not fully represent the spectrum of infections encountered in real-world clinical settings. Additionally, poor image quality may pose a challenge for accurate AI-assisted diagnosis, particularly for classifying healthy images. In future studies, we suggest the development of a preliminary DL model to exclude unfocused, poor-quality, or blank images commonly captured by IVCM technology. This model could assist in the diagnosis of clear images showing cellular structures and potentially improve the overall accuracy and speed of diagnosis.

Upon examining the misdiagnosed images, we found that the model may be influenced by hypo-reflectivity caused by interstitial edema and/or exudation, leading to the model overlooking inflammatory cells or pathogens. Additionally, hyper-reflective superficial epithelial cells can be misclassified as acanthamoeba cysts, and dendritic cells in the sub-basal layer of the epithelium, which are a hallmark of infectious keratitis such as AK^37^, may be mistaken for fungal filaments. However, such misdiagnoses are infrequent and can occur even among expert eye-care practitioners in clinical setting.

In conclusion, while the 96.93% accuracy of our proposed model may not be sufficient as a standalone diagnostic tool, it can still serve as a valuable reference for eye-care practitioners to make quicker and more confident diagnoses by leveraging the model’s predictions and saliency maps that highlight the probable infectious areas in the IVCM images. Moreover, this DL model could be integrated into the confocal scans software in a user-friendly fashion. We believe that future studies using larger IVCM datasets of more diverse infections from various institutions can further enhance model’s performance. To facilitate this effort, we have made the IVCM-Keratitis dataset openly available for future research. With more training data and more advanced techniques, models may still be able to improve further.

## Supplementary Information

Supplementary Information.

## Data Availability

The datasets generated during and/or analyzed during the current study are available in the Figshare repository, https://figshare.com/articles/dataset/Dataset/19838083.
