# Supplementary material for: Interpretable deep learning for diagnosis of fungal and acanthamoeba keratitis using in vivo confocal microscopy images
Source: Sci Rep. 2023 Jun 2;13:8953. doi: 10.1038/s41598-023-35085-9 (PMC10238502; doi:10.1038/s41598-023-35085-9)
Supplement: Supplementary file 1 — Supplementary Information. [file 41598_2023_35085_MOESM1_ESM.docx]

**Interpretable Deep Learning for Diagnosis of Fungal and Acanthamoeba Keratitis using In Vivo Confocal Microscopy Images**

Mahmoud Essalat^1^*^, †^, Mohammad Abolhosseini^2,3^*, Thanh Huy Le^4^*, Seyed Mohamadmehdi Moshtaghion^2,3^*, Mozhgan Rezaei Kanavi^2,3, †^

^1^ Department of Electrical and Computer Engineering, University of California, Los Angeles, California, USA

^2^ Ocular Tissue Engineering Research Center, Research Institute for Ophthalmology and Vision Science, Shahid Beheshti University of Medical Sciences, Tehran, Iran

^3^ Department of Confocal Scan, Central Eye Bank of Iran, Tehran, Iran

^4^ Department of Computer science, University of California, San Diego, California, USA

** These authors contributed equally to this work*

*† Corresponding authors*

† Correspondence to:

Mahmoud Essalat, PhD candidate,

Department of Electrical and Computer Engineering, University of California, Los Angeles

56-125B Engineering IV Building, UCLA, 420 Westwood Plaza, Los Angeles, CA 90095-1594, USA

Phone: (310) 825-2647

Email: [mahmoudessalat@ucla.edu](mailto:mahmoudessalat@ucla.edu)

Mozhgan Rezaei Kanavi, MD.

Ocular Tissue Engineering Research Center, Research Institute for Ophthalmology and Vision Science, Shahid Beheshti University of Medical Sciences, Tehran, Iran. No.23, Paidarfard St., Boostan 9 St., Pasdaran Ave., Tehran 1666673111, Iran

Phone: +98-21-2258-5952

E-mail: [rezaeikanavi@sbmu.ac.ir](mailto:rezaeikanavi@sbmu.ac.ir)

**Supplementary Table S1**. Detailed metrics of 10 different Deep Learning (DL) models for each class. Densenet161 showed the best performance among all models with an average weighted F1 score of 93.53%. and a F1 score of 94.04%, 91.69%, 97.28 and 90.95% for AK, FK, healthy, and NSK classes.

| Model | Stat | AK | FK | Healthy | NSK | Weighted Average |
| --- | --- | --- | --- | --- | --- | --- |
| Densenet161 | Accuracy | 95.70% | 96.50% | 98.90% | 95.90% | 96.93% |
|  | Sensitivity | 91.37% | 96.98% | 99.49% | 88.79% | 94.77% |
|  | Specificity | 98.25% | 96.38% | 98.75% | 98.05% | 97.80% |
|  | Precision | 96.86% | 86.94% | 95.17% | 93.21% | 92.52% |
|  | F1 | 94.04% | 91.69% | 97.28% | 90.95% | 93.53% |
| Densenet121 | Accuracy | 94.40% | 95.80% | 94.80% | 91.60% | 94.22% |
|  | Sensitivity | 88.68% | 97.49% | 74.24% | 91.81% | 87.79% |
|  | Specificity | 97.77% | 95.38% | 99.88% | 91.54% | 96.12% |
|  | Precision | 95.92% | 83.98% | 99.32% | 76.62% | 88.54% |
|  | F1 | 92.16% | 90.23% | 84.97% | 83.53% | 87.29% |
| Cspresnet50 | Accuracy | 91.10% | 93.70% | 93.00% | 90.00% | 92.15% |
|  | Sensitivity | 87.87% | 94.97% | 64.65% | 84.48% | 82.24% |
|  | Specificity | 93.00% | 93.38% | 100.00% | 91.67% | 94.85% |
|  | Precision | 88.11% | 78.10% | 100.00% | 75.38% | 85.47% |
|  | F1 | 87.99% | 85.71% | 78.53% | 79.67% | 82.42% |
| Resnet101 | Accuracy | 94.20% | 95.00% | 93.40% | 88.20% | 92.68% |
|  | Sensitivity | 90.30% | 95.48% | 66.67% | 84.91% | 83.48% |
|  | Specificity | 96.50% | 94.88% | 100.00% | 89.19% | 95.22% |
|  | Precision | 93.84% | 82.25% | 100.00% | 70.36% | 86.33% |
|  | F1 | 92.03% | 88.37% | 80.0% | 76.95% | 83.59% |
| Resnet152 | Accuracy | 92.80% | 93.60% | 97.50% | 92.10% | 94.25% |
|  | Sensitivity | 84.64% | 94.97% | 88.89% | 86.64% | 89.44% |
|  | Specificity | 97.62% | 93.26% | 99.63% | 93.75% | 95.95% |
|  | Precision | 95.44% | 77.78% | 98.32% | 80.72% | 87.38% |
|  | F1 | 89.71% | 85.52% | 93.37% | 83.58% | 88.01% |
| Resnext101 | Accuracy | 88.10% | 91.60% | 87.20% | 78.10% | 86.34% |
|  | Sensitivity | 84.37% | 76.88% | 35.35% | 81.47% | 66.94% |
|  | Specificity | 90.30% | 95.26% | 100.00% | 77.08% | 91.29% |
|  | Precision | 83.69% | 80.10% | 100.00% | 51.78% | 79.41% |
|  | F1 | 84.03% | 78.46% | 52.24% | 63.32% | 67.76% |
| Resnext50 | Accuracy | 90.76% | 92.47% | 94.58% | 87.25% | 91.51% |
|  | Sensitivity | 78.32% | 91.96% | 72.73% | 89.57% | 83.52% |
|  | Specificity | 98.09% | 92.60% | 100.00% | 86.55% | 94.13% |
|  | Precision | 96.01% | 75.62% | 100.00% | 66.67% | 83.79% |
|  | F1 | 86.27% | 82.99% | 84.21% | 76.44% | 82.22% |
| Vgg19 | Accuracy | 92.50% | 94.10% | 99.50% | 94.70% | 95.60% |
|  | Sensitivity | 95.42% | 81.91% | 97.47% | 83.62% | 83.62% |
|  | Specificity | 90.14% | 97.00% | 100.00% | 97.53% | 96.94% |
|  | Precision | 85.06% | 86.89% | 100.00% | 91.00% | 91.51% |
|  | F1 | 90.42% | 84.68% | 98.72% | 87.98% | 90.57% |
| Vgg16 | Accuracy | 91.60% | 92.80% | 98.80% | 93.60% | 94.59% |
|  | Sensitivity | 89.22% | 82.41% | 94.95% | 94.44% | 90.23% |
|  | Specificity | 93.00% | 95.38% | 99.88% | 95.57% | 96.38% |
|  | Precision | 88.27% | 81.59% | 99.47% | 85.59% | 88.94% |
|  | F1 | 88.74% | 82.0% | 96.89% | 86.32% | 88.56% |
| Vgg13 | Accuracy | 93.60% | 95.80% | 99.40% | 94.80% | 96.26% |
|  | Sensitivity | 89.49% | 91.46% | 98.48% | 90.09% | 92.88% |
|  | Specificity | 96.03% | 96.88% | 99.63% | 96.22% | 97.39% |
|  | Precision | 93.00% | 87.92% | 98.48% | 87.82% | 91.82% |
|  | F1 | 91.21% | 89.66% | 98.48% | 88.94% | 92.33% |


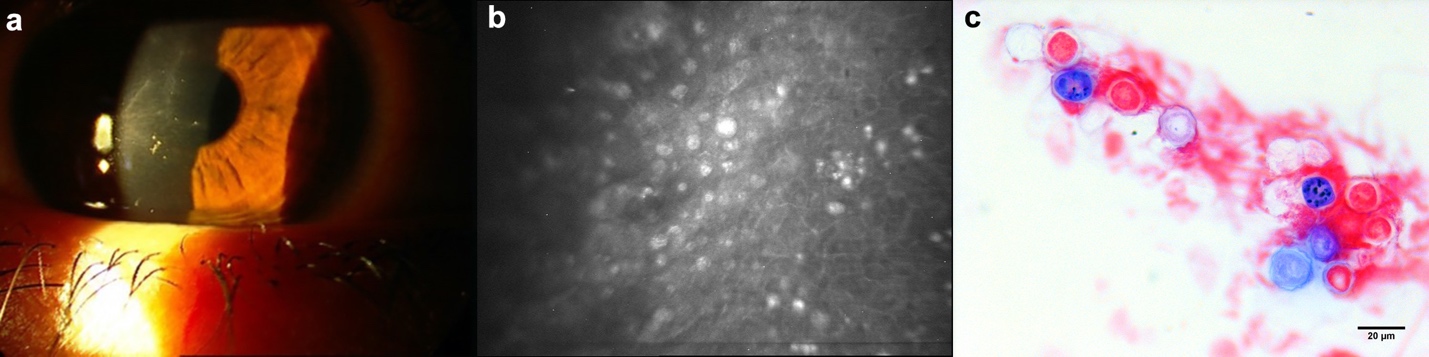


**Supplementary Figure S1.** Representative photoslit, confocal scan, and microbiological smear images from a patient with clinically suspected acanthamoeba keratitis. Note the presence of a superficial dendritic inflammation in the cornea (a) on slit-lamp biomicroscopy, scattered double-walled round and pear-shaped hyperreflective acanthamoeba cysts (b) on confocal scanning, and a few bilayered cystic structures of acanthamoeba in the gram-stained corneal smear (C).


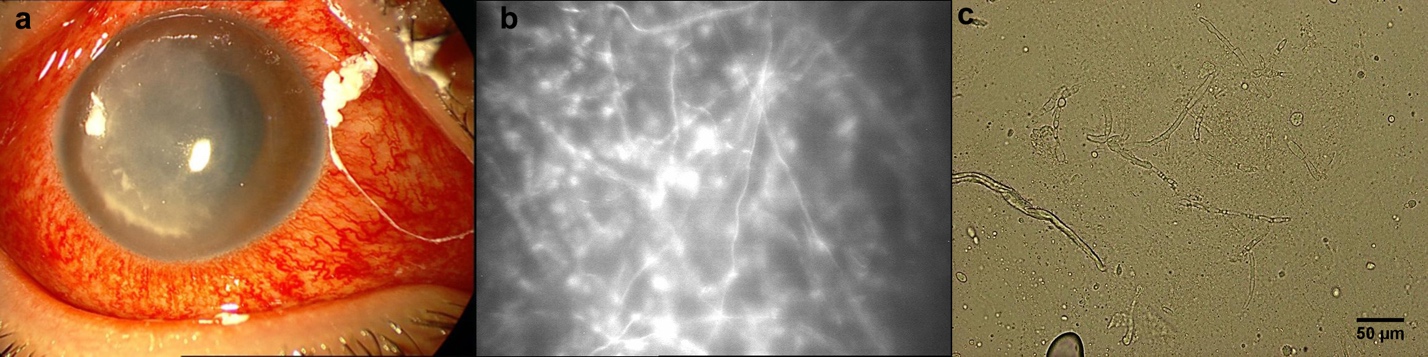

**Supplementary Figure S2.** Representative photoslit, confocal scan, and microbiological smear images from a patient with clinically suspected fungal keratitis. Note the presence of corneal inflammatory lesion with feathery margins on slit-lamp biomicroscopy (a), hyperreflective hyphal structures (b) on confocal scanning, and the presence of septate fungal hyphae on wet mount preparation of corneal smear (c).
